# SOX9 is a target of miR-134-3p and miR-224-3p in breast cancer cell lines

**DOI:** 10.1007/s11010-022-04507-z

**Published:** 2022-07-02

**Authors:** Tsu-Yang Chao, Theresa Kordaß, Wolfram Osen, Stefan B. Eichmüller

**Affiliations:** 1 GMP & T Cell Therapy Unit, German Cancer Research Center (DKFZ), 210, Im Neuenheimer Feld 280, D-69120, Heidelberg, Germany; 2 Faculty of Biosciences, University Heidelberg, Heidelberg, Germany

**Keywords:** miRNAs, SOX9, Breast cancer

## Abstract

The transcription factor SOX9 represents an important mediator of breast cancer progression. miRNAs are small non-coding RNAs inhibiting translation of target genes upon interaction with the 3′-UTR region of respective mRNA molecules. Deregulated miRNA expression is involved in hallmarks of cancer like sustained proliferation and inhibition of apoptosis. Here, we investigated the miRNA-mediated regulation of SOX9 expression in two breast cancer cell lines, thereby providing further insights into cellular mechanisms driving breast cancer progression. The modulating effects of miR-134-3p, miR-224-3p, and miR-6859-3p on SOX9 expression were analyzed by qPCR and Western blot in human MDA-MB-231 breast cancer cells. Direct binding of the above-mentioned miRNAs to the SOX9 3′-UTR was assessed by luciferase reporter assays and site-directed mutagenesis. Expression levels of the investigated miRNAs in tumor samples *versus* healthy tissues were analyzed in silico using publicly available databases. Transfection of miR-134-3p, miR-224-3p, or miR-6859-3p reduced SOX9 expression on mRNA and protein level. Reporter assays proved direct binding of miR-134-3p and miR-224-3p to the SOX9 3′-UTR in MDA-MB-231 and MCF-7 cells. Expression analysis performed in silico revealed reduced expression of both miRNAs in breast cancer tissues. We describe three novel miRNAs targeting SOX9 in human breast cancer cell lines. Among them miR-134-2p and miR-224-3p might act as tumor suppressors, whose down-regulation induces elevated SOX9 levels thereby promoting breast cancer progression.

**Supplementary Information:**

The online version contains supplementary material available at 10.1007/s11010-022-04507-z.

## Background

So far, 20 members of the SOX protein family have been described. Within this family, SRY-Box Transcription Factor 9 (SOX9) belongs to the SoxE subgroup of transcription factors which bind via HMG domains to specific DNA regions containing the AACAAT motif [1, 2]. SOX9 forms complexes with other SOX proteins or with further transcription factors such as members of the GLI zinc finger family. Complex formation is a prerequisite for targeted DNA binding of SOX9. Depending on its intracellular binding partners and the target site within the DNA, SOX9 can function either as a transcriptional repressor or activator [1]. For example, SOX9 was found highly expressed in various cancer types and thus associated with unfavorable clinical outcome [3]. Especially in breast cancer, SOX9 was identified as an important driver of cancer progression and has thus been referred to as “master regulator” of breast cancer cell fate [4]. Notably, SOX9 is highly expressed together with SOX4, 6, 8, 10 and 11 in triple-negative breast cancer, representing the breast cancer subtype with worst prognosis [5]. In line with these findings, silencing of SOX9 diminished viability of breast cancer cells and reduced invasion in vitro and in vivo by enhancing the expression of apoptosis related genes such as FADD, while decreasing the expression of genes involved in epithelial–mesenchymal transition like ZEB1 or CTNNB1 [5]. Furthermore, high expression of SOX9 has been described as a feature of cancer stem-like cells [6–8].

miRNAs belong to the group of small non-coding RNAs with a size of approximately 20 nucleotides, functioning as regulators of gene expression on post-transcriptional level. Argonaute proteins (AGO) are small RNA binding proteins involved miRNA processing and function. Among the four types of AGO proteins expressed in humans, AGO2 facilitates miRNA-mediated repression of translation by targeting respective mRNA molecules [9, 10]. Upon association with AGO proteins, miRNAs form the RNA-induced silencing complex (RISC) that binds via a short seed region of five to six nucleotides to the 3′-UTR region of the targeted mRNA molecule. Depending on the degree of complementary, this interaction will inhibit translation or cause degradation of the mRNA molecule [11–14]. Perfect base pairing between the miRNA and the targeted mRNA molecule activates the endonuclease activity of AGO2 resulting in cleavage of the target mRNA [14, 15]. Potentially, each miRNA can bind to several hundred different mRNA targets and each mRNA can be targeted by multiple miRNA species [14, 16, 17].

Generally, cancer cells exhibit aberrant miRNA expression patterns [18]. Thus miRNA expression profiling might gain clinical relevance, as miRNA expression patterns thus identified might resemble biomarkers in cancer therapy [18–21]. Understanding miRNA-mediated regulation of gene expression in cancer cells might provide deeper insights into mechanisms up-regulating expression of oncogenic genes, for example, by inhibited expression of tumor-suppressive miRNAs or through enhanced expression of oncogenic miRNAs (OncomiRs) [22, 23]. In this study we investigated how miRNAs can inhibit expression of the transcription factor SOX9, representing the “master regulator” of cell fate in breast cancer cells.

## Materials and methods

### Cell lines and cell culture

The human breast cancer cell lines MDA-MB-231 and MCF-7 were purchased from ATCC. HEK293 cells were provided by the DKFZ. MDA-MB-231 and HEK293 cells were cultured in RPMI 1640 medium (Gibco, Carlsbad, CA), while MCF-7 cells were cultured in DMEM medium at 37 °C and 5% CO_2_. Culture media supplemented with 10% FCS superior (Biochrom, Berlin, Germany) were used without antibiotics. Cell lines were regularly checked for mycoplasma contamination and cell line authenticity was verified by DNA fingerprinting.

### Transfection, RNA extraction and qPCR

Cells were cultured in 12-well plates until 70–80% confluency was achieved. Subsequently, cells were transfected with 50 nM miRNA using Lipofectamine RNAiMAX reagent (Thermo Fisher Scientific, Waltham, MA) according to the manufacturer’s protocol. Two days post transfection cells were harvested, and RNA was isolated using Qiagen RNeasy mini kit. RNA samples were measured using a Qubit™ 4 Fluorometer (Thermo Scientific, Boston, MA). A total of 500 ng RNA was reverse transcribed in a 20 μL reaction utilizing oligo(dT)18 primer using the Transcriptor First Strand cDNA Synthesis Kit (Roche Applied Science, Mannheim, Germany) according to the manufacturers protocol. All PCRs were performed in a Veriti 96 well Thermal Cycler (Applied Biosystem, Froster City, CA). qPCRs were performed with primers listed in Supplementary Table S3 and PowerUp SYBR Green Mastermix (Thermo Scientific, Boston, USA) utilizing a QuantStudio 3 Real-Time-PCR System (Thermo Scientific). The ribosomal protein L19 (RPL19) encoding gene was used as house-keeping gene. For all samples, three technical replicates were performed and relative expression was calculated with the 2^−ΔΔCT^ method relative to RPL19 according to the manufacturer’s protocol.

### Western blot

Cellular protein was isolated from frozen cell pellets by applying with 200 µL cell lysis buffer (Cell Signaling Technology, Cambridge, UK) supplemented with 1 mM phenylmethylsulfonylfluorid (PMSF). After 5 min incubation on ice, samples were centrifuged for 30 min at 13,000 rpm, 4 °C. Protein concentrations were measured using Qubit 4 Fluorometer (Thermo Scientific) and cell lysates were stored at -20 °C. Lysates were mixed with 5 × loading buffer and heated for 5 min at 95 °C to denature cellular protein. Subsequently, 20 µg protein were loaded per slot on a 12% polyacrylamide gel and separated by electrophoresis followed by electro-transfer onto a nitrocellulose membrane (Bio-Rad, Richmond, VA). After washing with TBS-T, the membrane was blocked for 1 h using 5% milk in TBS-T. The membrane was then incubated with the SOX9-specific primary antibody (AB5535, Sigma-Aldrich, St. Louis, MO) in 0.5% blocking solution in TBS-T at 4 °C overnight. After extensive washing (four times 5 min with TBS-T), the membrane was incubated with the respective horseradish peroxidase-conjugated secondary antibody (SC-2054, Santa Cruz Biotechnology, Heidelberg, Germany) for one hour at room temperature. After washing with TBS-T, protein bands were detected with an enhanced chemiluminescence (ECL) system (GE Healthcare, Buckinghamshire, UK) using a BioRad ChemiDoc XRS device. Densitometric quantification of protein bands was performed using ImageJ software. Actin was used as a positive control and the SOX9 protein level was normalized by division with actin protein expression levels. Full-size images of Western blots are depicted in Fig. S11.

### Luciferase-reporter assays

The pLS-SOX9 plasmid containing the SOX9 3′-UTR fused to the renilla luciferase reporter gene was purchased from Active motif (Active motif, La Hulpe, Belgium). In reporter assays, 100 ng pLS-vector were co-transfected with 50 nM miRNA using the DharmaFect Duo transfection reagent (GE Dharmacon, Lafayette, CO) according to the manufacturer’s protocol into cells seeded in 96-well cell culture plates on day prior to transfection. One day after transfection, luciferase signals were measured using the Lightswitch Luciferase Assay kit (Active Motif, La Hulpe, Belgium) according to the manufacturer´s protocol. Luminescence signal was assessed with CLARIOstar Plus reader (BMG LABTECH, Ortenberg, Germany). To verify direct binding, mutation of the miRNA binding site within the SOX9 3′-UTR was performed using the Quick-change mutagenesis II kit (Qiagen, Hilden, Germany) as outlined in the manufacturer’s protocol. Primers used for mutagenesis are listed in Table S1.

### Gene expression profiling

Gene expression profiling was performed on MDA-MB-231 cells transfected with mimic control-1, miR-134-3p, miR-224-3p, and miR-6859-3p. Therefore, 2 × 10^5^ cells/well were transfected with 50 nM miRNA in 12-well plates and 48 h post transfection RNA was isolated and sent to the DKFZ-Genomics and Proteomics Core Facility for microarray analysis using Affymetrix Clariom S human chip for all samples. Each condition was performed in triplicates. Processing of raw data was carried out by the Core Facility. Differential gene expression analysis was based on comparison to mimic control-1 samples. Venn diagrams were generated with webtool from Bioinformatics & Evolutionary Genomics Department of the Ghent University (http://bioinformatics.psb.ugent.be/webtools/Venn/). Gene-Set-Enrichment analysis was performed using the GSEA-MSigDB webtool (http://www.gsea-msigdb.org/gsea/index.jsp) [24, 25].

### Cell viability / proliferation assay

Cells were seeded in 96-well transparent culture plates to achieve 70–80% confluence on the day of transfection with 50 nM miRNAs or siRNAs (mimic control-1, miR-134-3p, miR-224-3p, miR-6859-3p). Cell viability assays were performed using the XTT dye (Cell Signaling Technology, Cambridge, UK). Therefore, 72 h post transfection cells were washed and incubated with XTT reagent at 37 °C for 1 h. Absorbance was measured at 450 nm using a ClarioStar Plus reader (BMG LABTECH, Ortenberg, Germany).

### Cell cycle analysis

MDA-MB-231 cells were seeded in 12-well plates and cultured until 70–80% confluency was reached on the day of transfection. Cells were transfected with 50 nM miRNA. After 72 h of incubation, the medium was discarded and the cells were washed with phosphate-buffered saline (PBS), detached with trypsin, and washed once with cold PBS. Then, 10 mL cold 70% ethanol was added dropwise to the cells while gentle vortexing. Preparations were stored at -20 °C for 48 h. At the day of cell cycle analysis using a FACS Canto II flow cytometry system (Becton Dickinson, Franklin Lakes, NJ), cells were washed three times with PBS, and stained with 100 µL of propidium iodide (PI) solution (50 µg/mL PI; 0.5 µg/mL RNase A) for 1 h at RT protected from light. Cell cycle stages were determined using FlowJo software.

### miRNA expression levels in breast cancer patients

Expression of miR-134 and miR-224 in breast cancer tissues of patients was assessed with the OncomiR Cancer data base [26]. Analysis of miR-134 and miR-224 expression was based on 87 samples from healthy tissue and 782 or 695 breast cancer derived samples, respectively. No data were available for miR-6859.

## Results

### miR-134-3p, miR-224-3p and miR-6859-3p are predicted regulators of SOX9 expression

Screening for miRNAs with known inhibitory effect on breast cancer cell proliferation and a seed sequence specific for the 3′-UTR of the SOX9 encoding mRNA, we determined miR-224-3p which had been published to inhibit proliferation of MDA-MB-231 cells [27]. Based on the webtool miRmap [28], the SOX9 3′-UTR was predicted to contain one binding site for the miR-224-3p seed region (see Supplementary Figure S1). Moreover, we identified miR-134-3p, whose expression was described to be down-regulated in breast cancer [29], while overexpression of this miRNA was reported to reduce breast cancer cell proliferation through targeting of apoptosis-inhibiting genes like BCL2 [30]. The 3′-UTR of the SOX9 mRNA exhibits one potential binding site for miR-134-3p (see Supplementary Figure S1), thus we selected miR-134-3p and miR-224-3p for further analysis as well as miR-6859-3p, the latter predicted to bind through its seed region to the SOX9 3′-UTR.

### miR-134-3p, miR-224-3p and miR-6859-3p mediated down-regulation of SOX9 expression is detectable on mRNA and protein level in MDA-MB-231 cells

**Fig. 1:**
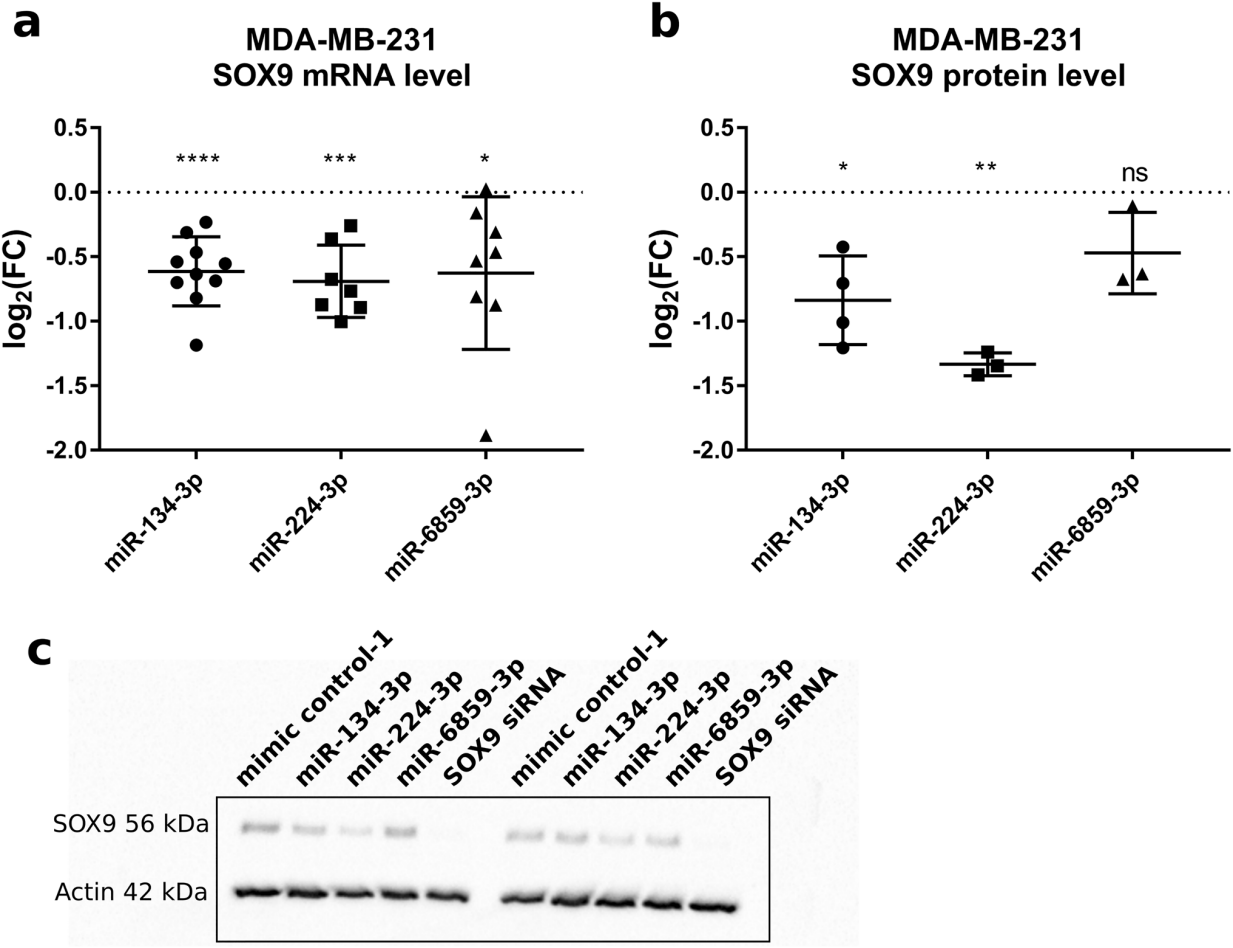
miR-134-3p, miR-224-3p and miR-6859-3p reduce SOX9 expression on mRNA and protein level in MDA-MB-231 cells. **A** MDA-BM-231 cells transfected with 50 nM miRNA cells were harvested 48 h later for RNA isolation and qPCR analysis. Fold changes in expression levels were calculated compared to mimic control-1 samples. RPL19 was used as house-keeping gene for qPCRs, since none of the tested miRNAs exhibits a binding site for this gene according to miRmap. For each miRNA, at least seven independent experiments were performed. **B** Western blot analysis performed with cellular protein isolated 72 h after transfection. Relative SOX9 protein expression was calculated compared to beta-actin as loading control, since none of the miRNAs carries predicted binding sites for the ACTB gene according to miRmap. **C** Quantification of protein levels was performed with ImageJ. One representative Western blot image is shown. At least three independent experiments were performed per condition. Fold changes were compared to mimic control-1 samples. Data is shown as Mean ± SD. Significance was assessed by one-sample T-test. *p < 0.05; **p < 0.01; ***p < 0.001; ****p < 0.0001

Next, we tested whether expression of the selected miRNAs could reduce intracellular SOX9 mRNA levels. Therefore, we selected the triple-negative breast cancer cell line MDA-MB-231 with sustained SOX9 expression. Two days after transfection with 50 nM miRNA we quantified SOX9 mRNA levels by qPCR. As depicted in Fig. 1A, all three miRNAs significantly reduced SOX9 mRNA levels, with miR-134-3p showing the most significant effect (mean log_2_(FC) = -0.61, *p* < 0.0001). Overall, miR-224-3p had the strongest effect on SOX9 mRNA levels (mean log_2_(FC) = -0.69, *p* = 0.0006). miR-6859-3p induced variable effects on SOX9 mRNA levels with log_2_(FC) fold changes ranging between -1.88 and 0.02. Still, miR-6859-3p reduced SOX9 mRNA levels to significant extent in this cell line (mean log_2_(FC) = -0.63, *p* = 0.02).

We then examined the impact of the selected miRNAs on SOX9 protein expression by Western blot analysis of transfected MDA-MB-231 cells. Consistent with the qPCR data, miR-224-3p mediated the strongest effect on SOX9 expression also on protein level (mean log_2_(FC) = -1.3, *p* = 0.0015) (Fig. 1B, C). Moreover, miR-134-3p also reduced SOX9 protein levels (mean log_2_(FC) = -0.84, *p* = 0.0166) significantly, whereas miR-6859-3p decreased SOX9 protein expression only to insignificant extent (mean log_2_(FC) = -0.47, ns).

### miR-134-3p and miR-224-3p function through direct interaction with the SOX9 3′-UTR in breast cancer cell lines.

**Fig. 2:**
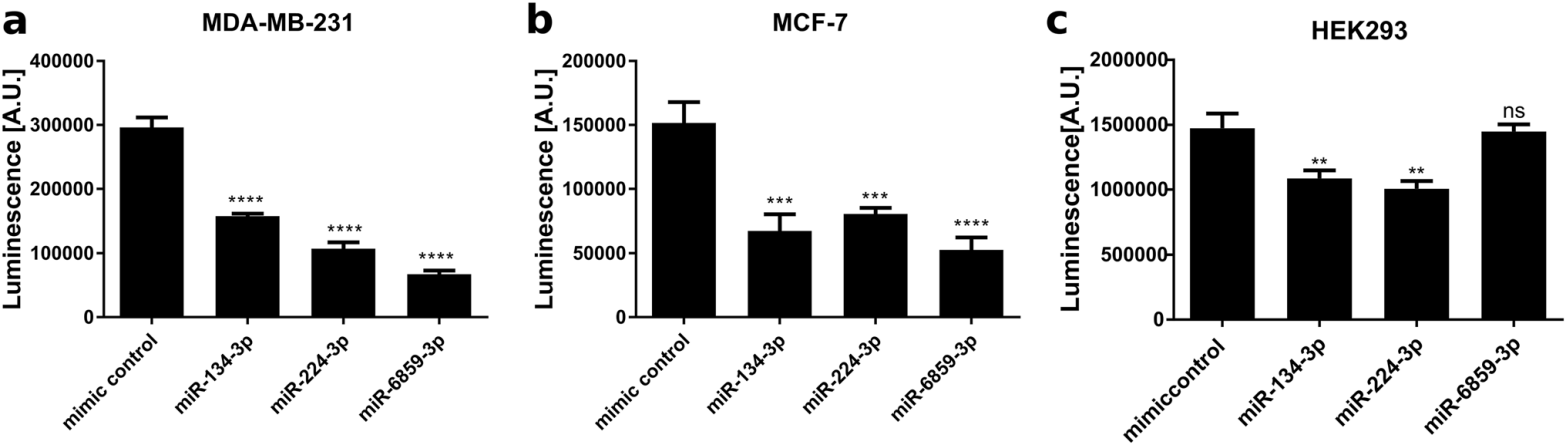
miR-134-3p and miR-224-3p directly bind to the SOX9 3′-UTR in breast cancer cell lines and in HEK293 cells. MDA-MB-231 (**A**), MCF-7 (**B**) or human embryonic kidney cells HEK293 (**C**) cells were transfected with 50 nM miRNA. Twenty-four hours later, luciferase activity was assessed by luminescence measurement. Per condition five individual transfections were performed. Data is shown as Mean ± SD. Significance was assessed by one-way ANOVA using Dunnett’s multiple comparison test. All samples were compared to mimic control-1 samples. *p < 0.05; **p < 0.01; ***p < 0.001; ****p < 0.0001

According to the miRmap tool, each of the miRNAs investigated has one binding site for the SOX9 3′-UTR. Thus, the observed inhibitory effects on SOX9 expression on mRNA and protein level are likely caused by direct interaction leading to a block in translation and to destabilization of SOX9 encoding mRNA. To prove direct interaction, we performed luciferase reporter assays using a pLS-SOX9 plasmid harboring the SOX9 3′-UTR fused to the renilla luciferase gene (Supplementary Figure S2). Thus, miRNA binding to the SOX9 3′-UTR would inhibit luciferase expression resulting in reduced luminescence signals. We co-transfected MDA-MB-231 cells with 50 nM miRNA and pLS-SOX9 vector and measured luminescence intensity 24 h post transfection. To exclude possible viability effects caused by the transfected miRNAs per se, we monitored viability of transfected MDA-MB-231 cells by XTT assays performed under the same conditions as applied during the reporter assay (see Supplementary Figure S3). We observed no effect on cell viability by the transfected miRNAs in this setting. Regarding direct binding of the miRNAs to the SOX3′-UTR we found that miR-6859-3p reduced the luciferase signal with pLS-SOX9 in MDA-MB-231 cells by 77% (*p* < 0.0001) (Fig. 2A). Also, miR-224-3p (*p* < 0.0001) and miR-134-3p (*p* = 0.0003) diminished the luminescence signal significantly by 64% and 47%, respectively. To determine, whether direct binding of miR-134-3p, miR-224-3p, and miR-6859-3p to the SOX9 3′-UTR was cell line independent, we repeated the luciferase assay with an additional breast cancer cell line, MCF-7 and with human embryonic kidney cells (HEK 293). In accordance with the effects observed in MDA-MB-231 cells, we confirmed decreased luminescence signal intensity in MCF-7 and in HEK293 cells to be caused by miR-134-3p and miR-224-3p (Fig. 2B, C), suggesting direct SOX9 targeting by these miRNAs across various breast cancer cell lines and HEK293 cells. Regarding miR-6859-3p, we noted a significant reduction of the luciferase signal in MCF-7 cells, however, this was not observed in HEK293 cells. An independent repetition of this assay with MDA-MB-231 and MCF-7 cells is shown in Supplementary Figure S4.

**Fig. 3:**
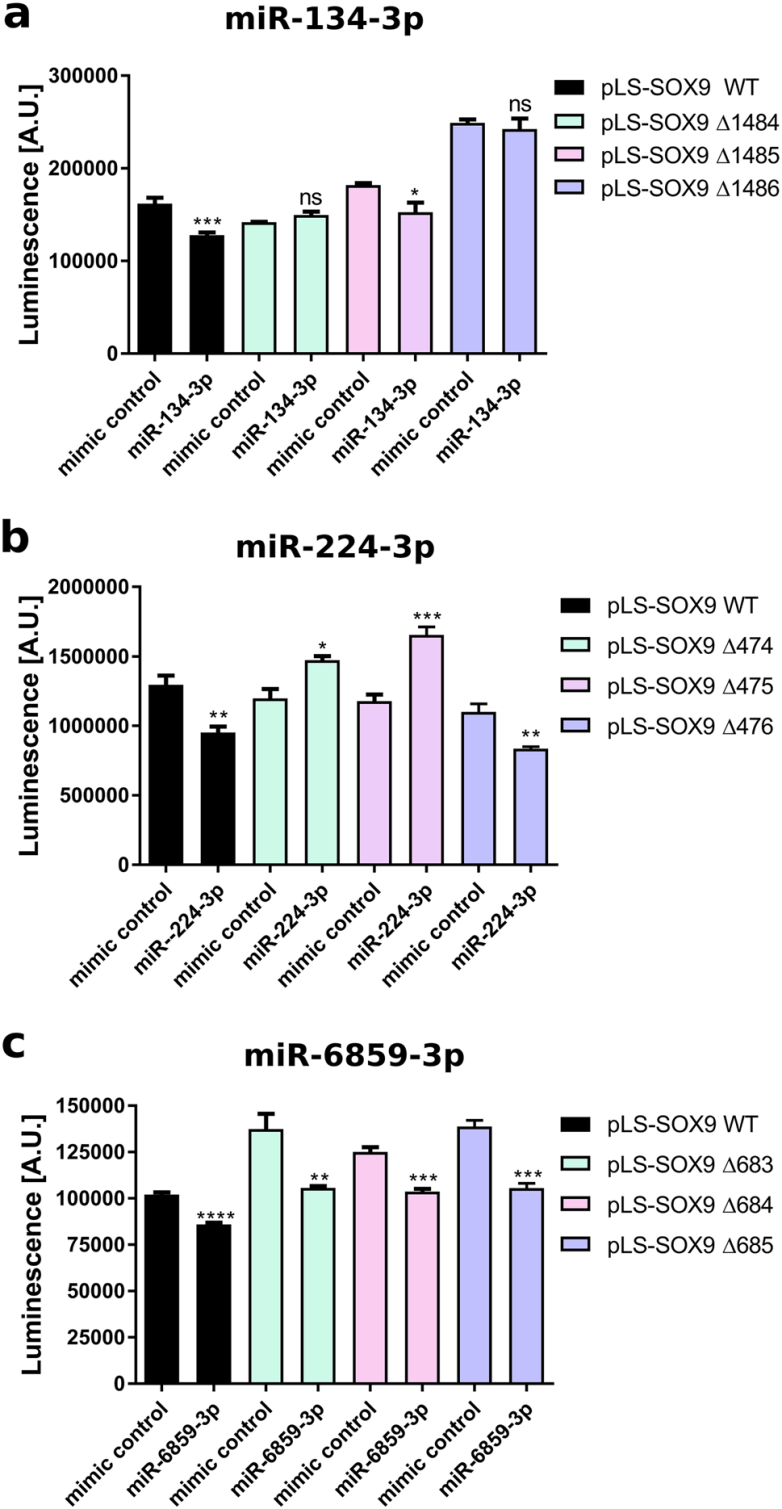
miR-134-3p and miR-224-3p, but not miR-6859-3p are direct binders of the SOX9 3′-UTR. MDA-BM-231 cells were co-transfected with 50 nM miRNA and luciferase encoding pLS-SOX9 constructs. Twenty-four hours later luciferase activity was quantified by luminescence measurement. **A** miR-134-3p mediated decrease in luciferase activity is abolished upon deletion of the nucleotides at positions 1484 or 1486, respectively. **B** Decreased luciferase activity mediated by miR-224-3p is reversed by deletion of the nucleotides at positions 474 or 475 within the SOX9 3′-UTR. **C** None of the deletions generated within the SOX9 3′-UTR restored the diminished luminesce signal caused by miR-6859-3p. For each condition five transfections were performed. Data is shown as Mean ± SD. Significance was assessed by one-way ANOVA using Dunnett’s multiple comparison test. All samples were compared to the respective mimic control samples. *p < 0.05; **p < 0.01; ***p < 0.001; ****p < 0.0001

Next, direct binding of the miRNA to the SOX9 3′-UTR was investigated through site-specific mutagenesis. Thus, we generated individual nucleotide deletions at three different positions within the respective targeting sites for miR-134-3p, miR-224-3p and miR-6859-3p of the SOX9 3′-UTR. The primers used to insert the deletions are listed in Supplementary Table S3. Upon transfection of miR-134-3p, a significant reduction of the luminescence activity was measured with the non-mutated plasmid, whereas deletions at position 1484 and 1486 restored the luciferase signal completely or close to the level of mimic control samples, respectively (Fig. 3A). miR-224-3p caused a significant reduction in luciferase signal with the non-mutated pLS-SOX9 vector, which is in line with the results shown in Fig. 2. Notably, deletion of positions 474 and 475, respectively, not only restored the luciferase signal, but even led to an increased signal compared to mimic control (Fig. 3B). Deletion at position 476 could not restore luciferase signal, and miR-224-3p had a significant inhibitory effect comparable to the wild-type plasmid. To analyze direct binding of miR-6859-3p, we used reporter constructs with deletions at the positions 683, 684 or 685 within the SOX9 3′-UTR. None of the deletions could restore the luciferase signal and the effects were still comparable to those observed after co-transfection of the construct containing the wild-type SOX9 3′-UTR (Fig. 3C).

In summary, we prove direct binding of miR-134-3p and miR-224-3p to the SOX9 3′-UTR as underlying mechanism of their repressive effects on SOX9 expression. However, the observed effects of miR-6859-3p on SOX9 expression were most likely caused by indirect mechanisms.

### miR-134-3p and miR-224-3p affect cell cycle processes in MDA-MB-231 cells

**Fig. 4:**
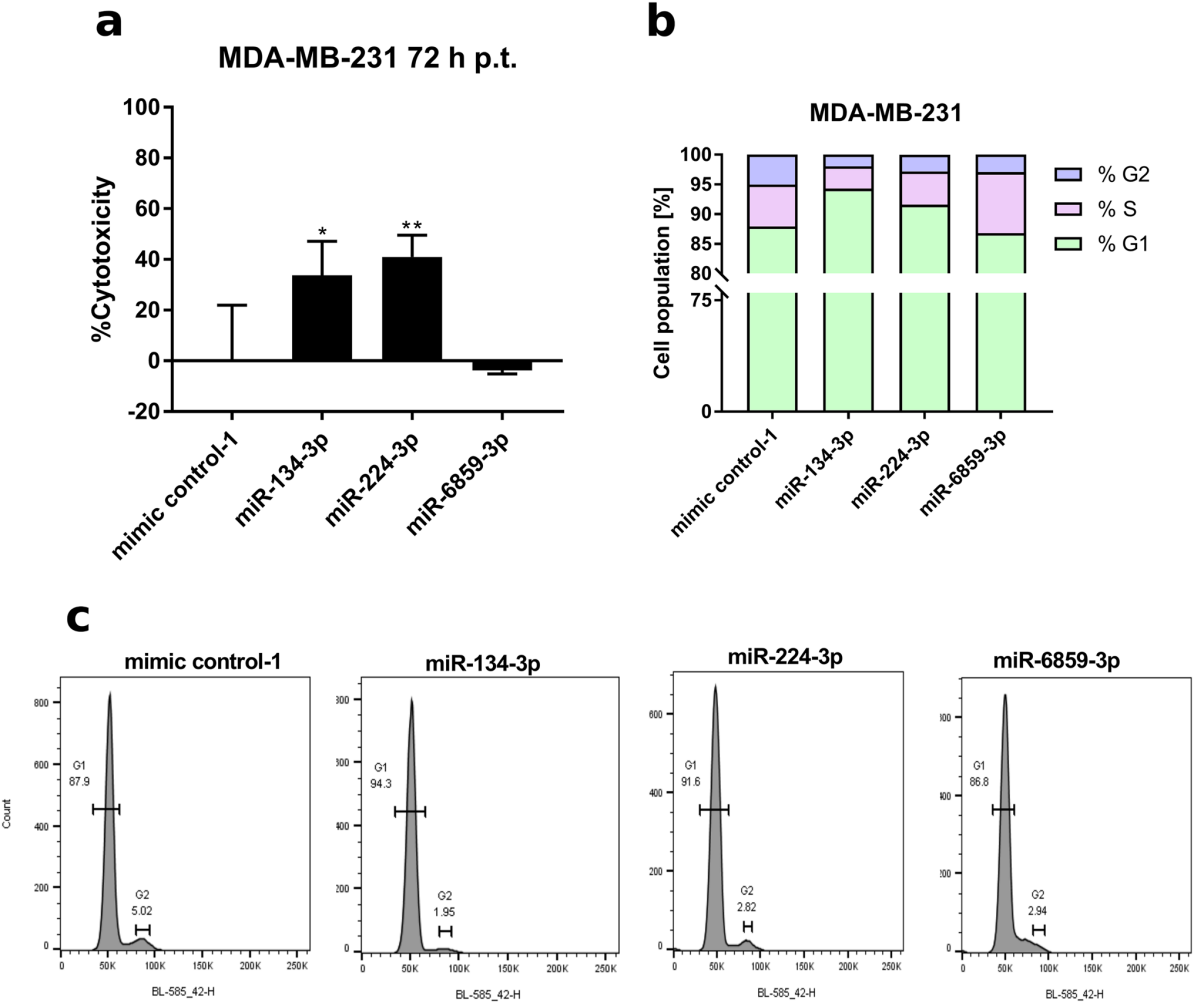
Overexpression of miR-134 or miR-224 in MDA-BM-231 cells decreases viability and enhances the proportion of cells in G1 phase. MDA-BM-231 cells were transfected with 50 nM miRNA and cell viability as well as cell cycle stages were assessed 72 h post transfection by XTT assay **(A)** or flow cytometry **(B, C)**, respectively. Four transfections were performed per condition for XTT assays. Data is shown as Mean ± SD. Significance was assessed by one-way ANOVA using Dunnett’s multiple comparison test. All samples were compared to the respective mimic control-1 samples. *p < 0.05; **p < 0.01; ***p < 0.001; ****p < 0.0001

To determine the overall effects of miR-134-3p, miR-224-3p and miR-6859-3p in MDA-MB-231 cells, we performed gene expression profiling 48 h after individual miRNA transfection. Figure 4 shows the overlap of genes significantly down-regulated by these three miRNAs. The impact on SOX9 expression was significant as well (see Supplementary Figure S5); however, the fold change of SOX9 expression, especially upon miR-6859-3p transfection, did not range among the maximally affected genes. Genes collectively down-regulated (FC < 0.5) were selected for subsequent Gene-Set-Enrichment analysis. The enriched GO-terms and the corresponding genes are listed in Supplementary Table S4. In particular, pathways related to chromatin and its organization were enriched. Overall, miR-224-3p and miR-134-3p showed higher similarity in their down-regulating expression pattern and shared 39 strongly decreased genes (Supplementary Figure S6). We also performed pathway enrichment analysis on the genes exclusively inhibited by miR-134-3p and miR-224-3p, neglecting miR-6859-3p. These results are summarized in Supplementary Table S5. Here, many GO-terms related to cell cycle, DNA repair and replication were significantly enriched. We also analyzed genes commonly up-regulated upon miRNA transfection (Supplementary Figure S7). Only the following four genes, RHOB, CHRNA1, MYO5B and TRIM21 showed significantly enhanced expression (FC > 2) upon transfection with miR-134-3p and miR-224-3p, and no enriched pathways were determined for these genes. RHOB is involved in apoptotic processes following DNA damage [31] and expression of RHOB was strongly increased upon transfection of miR-134-3p (FC = 3.2) and miR-224-3p (FC = 4.8), respectively. miR-6859-3p enhanced RHOB mRNA levels as well, albeit to lower extent (FC = 2.1).

Since our microarray data pointed towards an inhibitory function of miR-134-3p and miR-224-3p on cell cycle and proliferation, we performed cell viability assays and cell cycle analyses in transfected MDA-MB-231 cells. miR-134-3p and miR-224-3p significantly decreased the cell viability of MDA-MB-231 cells 72 h post transfection, whereas miR-6859-3p had no effect on viability compared to mimic control transfections (Fig. 4A). Regarding cell cycle analysis, transfection of miR-134-3p, miR-6859-3p and miR-224-3p resulted in a smaller percentage of cells within G2 phase (%G2_miR-134_ = 1.95, %G2_miR-6859_ = 2.94, %G2_miR-224_ = 2.82) compared to mimic control transfection (%G2_control_ = 5.02). However, transfection with miR-134-3p or miR-224-3p led to a higher proportion of cells in G1 phase (%G1_miR-134_ = 94.3, %G1_miR-224_ = 91.6, %G1_control_ = 87.9). The proportion of cells in G1 phase after miR-6859-3p transfection was similar to the control (%G1_miR-6859_ = 86.8). Notably, upon miR-6859-3p transfection, twice as many cells were in S phase (%S_miR-6859_ = 10.26) compared to control.

### Expression of miR-134-3p and miR-224-3p is reduced in breast cancer samples

**Fig. 5:**
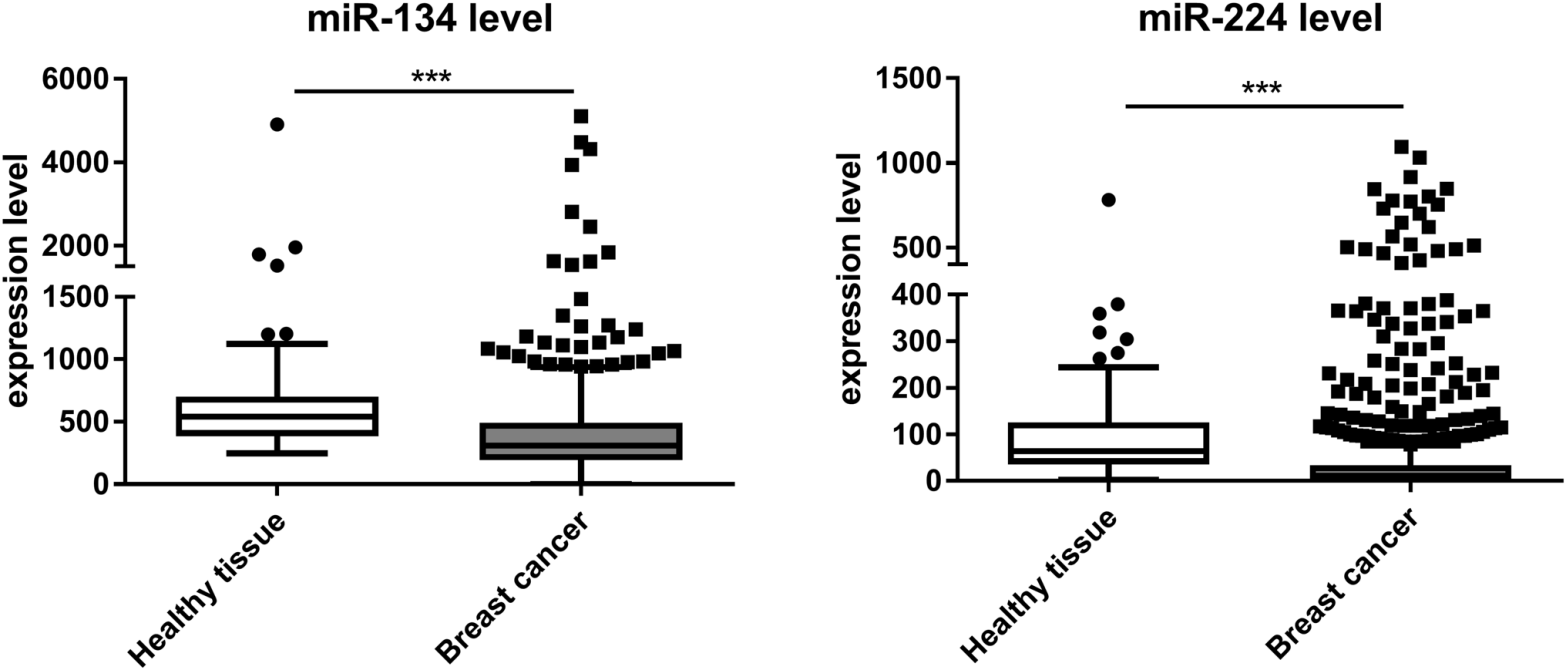
miR-134 and miR-224 expression levels in healthy tissue vs. breast cancer samples. Expression levels of miR-134 and miR-224 in tissue samples from healthy donors and breast cancer patients were compared. Both miRNAs are expressed at significantly lower level in breast cancer samples compared to healthy breast tissue. Analysis of miR-134 and miR-224 expression was based on 87 samples from healthy tissue and 782 or 695 breast cancer derived samples, respectively. No data were available for miR-6859. Tukey-style box-and-whiskers plots were used to present the data. Median expression level is indicated. Significance was assessed by unpaired T-test with Welch correction. ***p < 0.001

We then assessed the clinical relevance of the investigated miRNAs and compared their expression levels in breast cancer specimens and healthy breast tissue applying the OncomiR Cancer data base. As a result, miR-134 and miR-224 expression levels were found to be significantly decreased in breast cancer samples compared to healthy tissue, as shown in Fig. 5. Overall, miR-134 levels were higher than those of miR-224. The mean expression for miR-134 was 40% lower compared to healthy tissue (Normal_miR134_ = 652, BC_miR134_ = 406, *p* = 0.0001) and the level of miR-224 level was reduced by 45% compared to normal tissue (Normal_miR224_ = 104, BC_miR224_ = 57, *p* = 0.0005). For miR-6859 no expression data were available.

## Discussion

This paper shows for the first time the inhibitory effect of miR-134-3p, miR-224-3p and miR-6859-3p on SOX9 expression on transcriptional and translational level in human breast cancer cell lines. Although miR-6859-3p had the lowest impact on SOX9 expression, this miRNA showed inhibitory effects in reporter assays in both breast cancer cell lines tested, but not in HEK293 cells. miR-6859-3p was predicted to bind to the SOX9 3′-UTR, however, we were unable to prove direct binding in our assays with constructs containing site-specific point mutations. This strongly suggests that miR-6859-3p dependent repression of SOX9 expression is mediated indirectly, offering an explanation for the different results observed with miR-6859-3p in the luciferase assays performed with the breast cancer cell lines and HEK293 cells, respectively, pointing towards a cell type specific function of this miRNA.

Regarding miR-224-3p and miR-134-3p we could prove direct binding to the SOX9 3′-UTR as underlying mechanism of their inhibitory effect on SOX9 expression. Furthermore, transfection of miR-134-3p and miR-224-3p, respectively, reduced the viability of MDA-MB-231 breast cancer cells, and gene expression profiling confirmed the impact of these miRNAs on cell cycle associated pathways. Considering their suppressive effects on expression of SOX9 as master regulator of cell fate in breast cancer and their inhibitory impact on the breast cancer cell viability, miR-224-3p and miR-134-3p could be considered as potential tumor-suppressive miRNAs in breast cancer. This is in line with the OncomiR Cancer data base documenting reduced expression levels of these miRNAs in breast cancer tissues. Regarding miR-6859-3p, more experiments are needed to determine, whether this miRNA classifies as a tumor-suppressive miRNA. Interestingly, miR-134-3p has been described by others to inhibit cancer progression. For example, it was demonstrated that miR-134-3p overexpression lowered ovarian cancer cell proliferation and caused cell cycle arrest as well as inhibition of migration and invasion [32]. With respect to ovarian cancer, the effects were partially caused by down-regulated expression of FEN1 mediated by miR-134-3p [32]. Indeed, a tumor-suppressive function of miR-134 was also shown in small-cell lung cancer. In the study conducted by Qin et al*.,* inhibited proliferation caused by miR-134 expression was partly mediated through direct targeting of ITGB1 [33]. These findings are in accordance with our results demonstrating that miR-134-3p might be considered as tumor-suppressive miRNA in breast cancer, ovarian and small-cell lung cancer. However, further functional in vitro and in vivo studies, are necessary to verify this notion. In contrast, oncogenic features of miR-134 have been described for other cancer types, thus whether miR-134 causes cancer progression or suppression might depend on the tumor entity [34].

In osteosarcoma it has been described, that the long non-coding RNA (lncRNA) SNHG4 enhances cancer progression by sponging miR-224-3p. The cancer-promoting effects of SNHG4 could be reversed by miR-224-3p overexpression [35]. Conversely, in non-small lung cancer it was found that sponging of miR-224-3p by the lncRNA HCG11 enhances cancer cell proliferation and inhibits apoptosis [36]. However, our findings together with previous data obtained from studies in breast cancer [27] support a possible tumor-suppressive role of miR-224-3p.

**Fig. 6:**
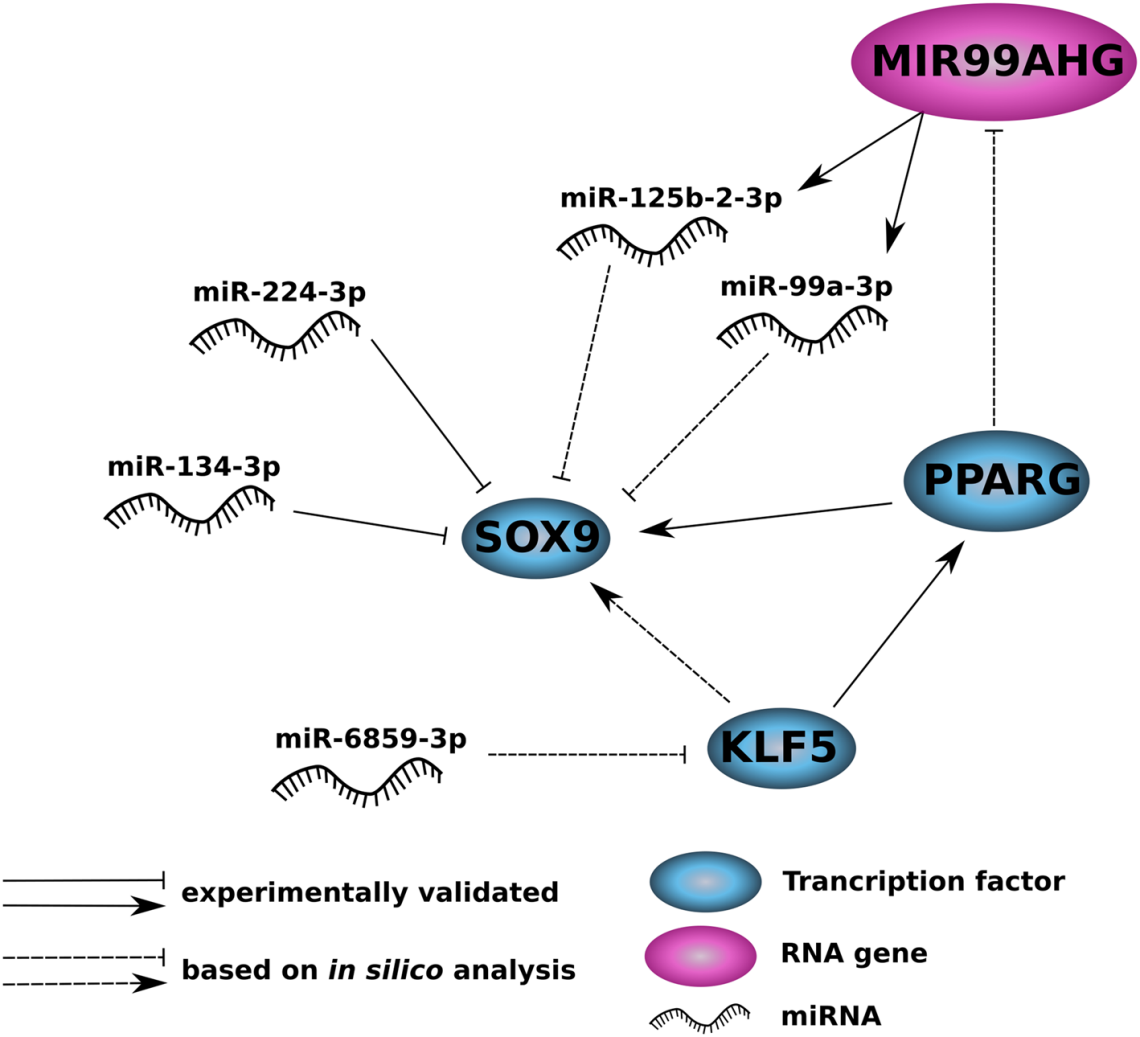
Graphical summary of the study. miR-134-3p and miR-224-3p were shown to inhibit SOX9 expression by direct binding to the SOX9 3′-UTR. miR-6859-3p might inhibit SOX9 expression at protein and mRNA level through indirect mechanisms, i.e., independently from binding to the SOX9 3′-UTR. Expression of the MIR99AHG gene encoding miR-125b-2-3p and miR-99a-3p, both carrying a binding site for SOX9 3′-UTR, was up-regulated after miR-6859-3p transfection in MDA-MB-231 cells. The transcription factors KLF5 and PPARG were significantly down-regulated upon miR-6859-3p transfection. We propose that miR-6859-3p might inhibit KLF5 expression directly, resulting in reduced levels of PPARG, which under normal conditions might suppress MIR99AHG transcription

Seeking explanations why disruption of the miR-6859-3p binding site within the SOX9 3′-UTR had no effect in our reporter assays, we focused on the predicted secondary RNA structure (Supplementary Figure S8). Interestingly, while the putative binding sites for miR-134-3p and miR-224-3p are located in unpaired regions, the predicted binding site for miR-6859-3p is found in a paired region within a loop structure, suggesting that binding of miR-6859-3p within SOX9 3′-UTR was prevented by structural hindrance. We therefore conclude that miR-6859-3p suppresses SOX9 expression indirectly, e.g., by repressing a transcriptional activator of SOX9 or by altering the endogenous miRNA expression pattern. Interestingly, we found expression of the MIR99AHG gene, encoding the miR-99a/let-7c/miR-125b-2 miRNAs, significantly enhanced in MDA-MB-231 cells following miR-6859-3p transfection. Both, miR-99a-3p and miR-125b-2-3p have a binding site for the SOX9 3′-UTR (see Supplementary Figure S9). Based on our microarray data and on correlation analysis using the NCI-60 data set [37], we identified PPARG as a transcription factor whose expression was negatively correlated with MIR99AHG mRNA levels and furthermore, appeared repressed upon miR-6859-3p transfection (see Supplementary Figure S10). Indeed, the MIR99AHG promoter region contains a PPARG binding site, hence this transcription factor might function as a transcriptional repressor of the MIR99AHG gene. Since miR-6859-3p is not a predicted binder of the PPARG 3′-UTR, we looked for transcription factors significantly reduced in miR-6859-3p transfected MDA-MB-231 cells. We found KLF5, a known transcriptional activator of PPARG [38, 39], to be repressed by miR-6859-3p transfection. Since miR-6859-3p carries a predicted binding site for the KLF5 3′-UTR, this miRNA might inhibit KLF5 expression resulting in decreased PPARG levels. As a consequence, MIR99AHG expression as well as endogenous levels of miR-99a-3p and miR-125b-2-3p would be enhanced. The latter miRNAs potentially block SOX9 expression via binding to the SOX9 3′-UTR, possibly explaining the reduced signals observed in the luciferase reporter assay with the two miR-6859-3p transfected breast cancer cell lines. In fact, HEK293 cells exhibit low basal expression levels of KLF5, which might account for the different effects detected among the breast cancer cell lines and HEK293 cells. Interestingly, the SOX9 promoter contains a potential KLF5 binding site and KLF5 expression is indeed positively correlated with SOX9 mRNA levels (see Supplementary Figure S10). However so far, this interaction has not been validated experimentally. The SOX9 promoter contains also a binding site for PPARG and in fact, increased SOX9 levels were observed in HCT-116 cells upon treatment with the PPARG activator rosiglitazone [40]. Thus, miR-6859-3p might inhibit transcriptional activation of SOX9 through reduction of KLF5 levels and further downstream also of PPARG levels. Additionally, miR-6859-3p mediated inhibition of KLF5 expression might enhance expression of the SOX9 targeting miRNAs miR-99a-3p and miR-125b-2-3p indirectly. This hypothesis together with the findings of our study are illustrated in Fig. 6.

## Conclusion

We demonstrate that miR-134-3p, miR-224-3p, and miR-6859-3p diminish SOX9 expression in human breast cancer cells. This finding might help to understand breast cancer specific up-regulation of SOX9 expression accompanied by enhanced tumor cell proliferation and increased tumor growth. Down-regulation of miR-134-3p and miR-224-3p expression might represent an initial event in breast cancer development, leading to elevated SOX9 levels that will drive breast cancer progression.

## Supplementary Information

Below is the link to the electronic supplementary material.

Supplementary file1 (DOCX 3336 KB)

## Data Availability

Original data are stored at the senior author’s lab and can be provided upon request.

## Abbreviations

AGO: Argonaute protein
FC: Fold change
lncRNA: Long non-coding RNA
miRNA: MicroRNA
OncomiR: Oncogenic miRNA
PCC: Pearson’s Correlation Coefficient
RISC: RNA-induced silencing complex
RPL19: Ribosomal Protein L19
SOX9: SRY-Box Transcription Factor 9
UTR: Untranslated region
