# Supplementary material for: SOX9 is a target of miR-134-3p and miR-224-3p in breast cancer cell lines
Source: Mol Cell Biochem. 2022 Jul 2;478(2):305–15. doi: 10.1007/s11010-022-04507-z (PMC9886654; doi:10.1007/s11010-022-04507-z)
Supplement: Supplementary file 1 — Supplementary file1 (DOCX 3336 KB) [file 11010_2022_4507_MOESM1_ESM.docx]

Supplemental Material to the paper:

SOX9 is a target of miR-134-3p, miR-224-3p and miR-6859-3p in breast cancer cell lines

by Tsu-Yang Chao^*^, Theresa Kordaß^*^, Wolfram Osen, and Stefan B. Eichmüller

**Supplementary Materials and Methods**

**miRNAs and siRNAs**

All miRNAs (MISSION miRNA mimics) were purchased from Sigma-Aldrich (St. Louis, USA). The siRNA pool against SOX9 was purchased from Dharmacon/Horizon Discovery (Lafayette, USA).

## XTT proliferation assay and luciferase assay conditions

## Cells were seeded in 96-well transparent culture plates to achieve 70-80 % confluence on the day of transfection. After 24 hours, cells were transfected with 50 nM miRNAs or siRNAs (mimic control-1, miR-134-3p, miR-224-3p, miR-6859-3p, SOX9 siRNA pool). Proliferation assays were performed using XTT dye. Cells were washed and incubated with XTT reagent in the incubator for 6 hours. The spectro-photometrical absorbance of the samples was measured using a microplate (ELISA) reader. The wavelength to measure the absorbance of the formazan product is 450 nm.

**Supplementary Tables**

**Table S1.** Antibodies used for FACS and Western blot analysis.

| **Antibody** | **Type** | **Manufacturer** |
| --- | --- | --- |
| Mouse anti-human actin | clone C4 | MP Biomedicals, Illkirch,  France |
| Goat anti-mouse sc2005 | polyclonal | Santa Cruz Biotechnology,  Heidelberg, Germany |
| Goat anti-rabbit sc2004 | polyclonal | Santa Cruz Biotechnology,  Heidelberg, Germany |
| Rabitt anti-human SOX9 AB5535 | polyclonal | Sigma-Aldrich, St. Louis, USA |

**Table S2.** Sequence of miRNAs and siRNAs used in this study.

| **miRNA** | **Sequence 5’-3’** | **Purpose** |
| --- | --- | --- |
| **hsa-miR-134-3p** | CCUGUGGGCCACCUAGUCACCAA | candidate miRNA |
| **hsa-miR-224-3p** | AAAAUGGUGCCCUAGUGACUACA | candidate miRNA |
| **hsa-miR-6859-3p** | UGACCCCCAUGUCGCCUCUGUAG | candidate miRNA |
| **mimic control-1** | GGUUCGUACGUACACUGUUCA | non-targeting control miRNA |
| **SOX9 siRNA pool ON Target plus SMARTpool** | GGAACAACCCGUCUACACA GAACAAGCCGCACGUCAAG GACCUUCGAUGUCAACGAG GGAAGUCGGUGAAGAACGG | GE Healthcare Dharmacon, Freiburg, Germany |

**Table S3.** Primers used in this study. BS = binding site in SOX9 3’-UTR.

| **miRNA** | **Sequence 5’-3’** | **Purpose** |
| --- | --- | --- |
| **RPL19_fwd** | GGCACATGGGCATAGGTAAG | qPCR |
| **RPL19_rev** | CCATGAGAATCCGCTTGTTT | qPCR |
| **SOX9_fwd** | AGCGAACGCACATCAAGAC | qPCR |
| **SOX9_rev** | CTGTAGGCGATCTGTTGGGG | qPCR |
| **del_1484_fwd** | TTTGCAGTGTTTTCTGCACAGACCTTTGGGCTGC | mutation miR-134-3p BS |
| **del_1484_rev** | GCAGCCCAAAGGTCTGTGCAGAAAACACTGCAAA | mutation miR-134-3p BS |
| **del_1485_fwd** | GCAGTGTTTTCTGCCCAGACCTTTGGGCTG | mutation miR-134-3p BS |
| **del_1485_rev** | CAGCCCAAAGGTCTGGGCAGAAAACACTGC | mutation miR-134-3p BS |
| **del_1486_fwd** | GTCATTTGCAGTGTTTTCTGCCAAGACCTTTGGGCT | mutation miR-134-3p BS |
| **del_1486_rev** | AGCCCAAAGGTCTTGGCAGAAAACACTGCAAATGAC | mutation miR-134-3p BS |
| **del_474_fwd** | TTTTAGTATGTACTGTGTATGATTCATTACATTTTGAGGGGATTTATACATATTTTTAG | mutation miR-224-3p BS |
| **del_474_rev** | CTAAAAATATGTATAAATCCCCTCAAAATGTAATGAATCATACACAGTACATACTAAAA | mutation miR-224-3p BS |
| **del_475_fwd** | GTATGTACTGTGTATGATTCATTACCTTTTGAGGGGATTTATACATATTTTT | mutation miR-224-3p BS |
| **del_475_rev** | AAAAATATGTATAAATCCCCTCAAAAGGTAATGAATCATACACAGTACATAC | mutation miR-224-3p BS |
| **del_476_fwd** | GTATGTACTGTGTATGATTCATTACCATTTGAGGGGATTTATACATATTTTTAG | mutation miR-224-3p BS |
| **del_476_rev** | CTAAAAATATGTATAAATCCCCTCAAATGGTAATGAATCATACACAGTACATAC | mutation miR-224-3p BS |
| **del_683_fwd** | TGTGTTATGTGATCAGTTTTGGGGTTAACTTTGCTTAATTCCTC | mutation miR-6859-3p BS |
| **del_683_rev** | GAGGAATTAAGCAAAGTTAACCCCAAAACTGATCACATAACACA | mutation miR-6859-3p BS |
| **del_684_fwd** | GTGTTATGTGATCAGTTTTGGGGTTAACTTTGCTTAATTCCTCA | mutation miR-6859-3p BS |
| **del_684_rev** | TGAGGAATTAAGCAAAGTTAACCCCAAAACTGATCACATAACAC | mutation miR-6859-3p BS |
| **del_685_fwd** | TGTTATGTGATCAGTTTTGGGGTTAACTTTGCTTAATTCCTCAG | mutation miR-6859-3p BS |
| **del_685_rev** | CTGAGGAATTAAGCAAAGTTAACCCCAAAACTGATCACATAACA | mutation miR-6859-3p BS |

**Table S4.** GSEA analysis of genes commonly down-regulated by miR-134-3p, miR-224-3p and miR-6859.3p (FC < 0.5).

| **Gene Set name** | **Genes in overlap** | **FDR q-value** |
| --- | --- | --- |
| **DNA packaging complex** | H2AC16, H2AC17, H2AC21, H2AC19, H2BC9, H2BC11, H2BC13 | 1.18 e-13 |
| **Protein DNA complex** | H2AC16, H2AC17, H2AC21, H2AC19, H2BC9, H2BC11, H2BC13 | 3.57 e-12 |
| **Protein heterodimerization activity** | H2AC16, H2AC17, H2AC21, H2AC19, H2BC9, H2BC11, H2BC13 | 5.48 e-11 |
| **Chromatin organization** | H2AC16, H2AC17, H2AC21, H2AC19, H2BC9, H2BC11, H2BC13 | 2.51 e-8 |
| **Protein dimerization activity** | H2AC16, H2AC17, H2AC21, H2AC19, H2BC9, H2BC11, H2BC13 | 1.13 e-7 |
| **Chromatin** | H2AC16, H2AC17, H2AC21, H2AC19, H2BC9, H2BC11, H2BC13 | 2.97 e-7 |
| **Chromosome organization** | H2AC16, H2AC17, H2AC21, H2AC19, H2BC9, H2BC11, H2BC13 | 2.97 e-7 |
| **Chromatin silencing** | H2AC16, H2AC17, H2AC21, H2AC19 | 1.09 e-6 |
| **Chromosome** | H2AC16, H2AC17, H2AC21, H2AC19, H2BC9, H2BC11, H2BC13 | 2.84 e-6 |
| **Negative regulation of gene expression epigenetic** | H2AC16, H2AC17, H2AC21, H2AC19 | 6.22 e-6 |

**Table S5.** GSEA analysis of genes down-regulated by miR-134-3p and miR-224-3p but not miR-6859-3p. (FC < 0.5).

| **Gene Set name** | **Genes in overlap** | **FDR q-value** |
| --- | --- | --- |
| **Chromosome organization** | 19 | 1.76 e-17 |
| **DNA metabolic process** | 17 | 1.32 e-16 |
| **Chromosome** | 20 | 1.32 e-16 |
| **Cell cycle** | 18 | 2.27 e-13 |
| **DNA replication** | 11 | 2.27 e-13 |
| **DNA conformation change** | 11 | 2.37 e-12 |
| **DNA repair** | 11 | 3.84 e-10 |
| **Cell cycle process** | 14 | 7.81 e-10 |
| **Telomere organization** | 8 | 8.4 e-10 |
| **Cellular response to DNA damage** | 12 | 9.22 e-10 |

**
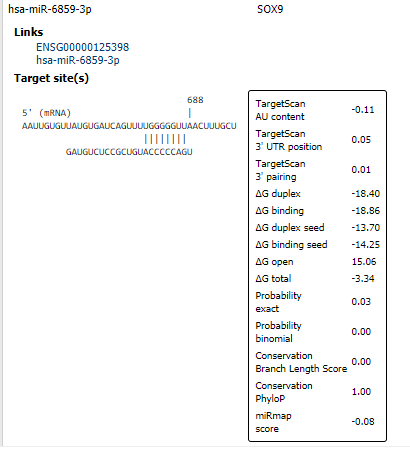
Supplementary Figures**

**
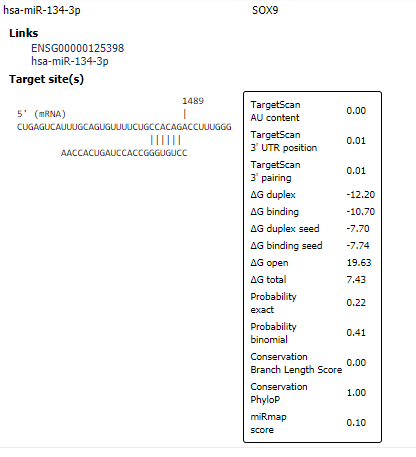

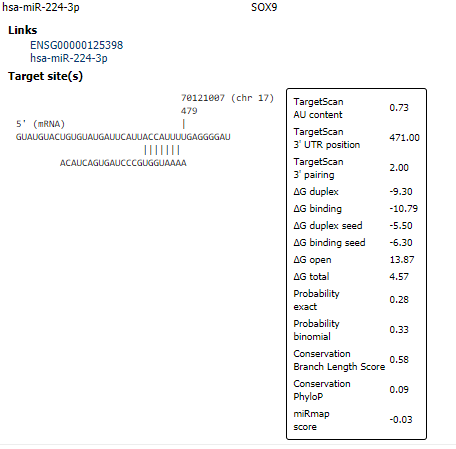
**

**Fig. S1. miRNA binding sites within SOX9 3’-UTR.** Binding sites were retrieved with miRmap tool (https://mirmap.ezlab.org/app/).


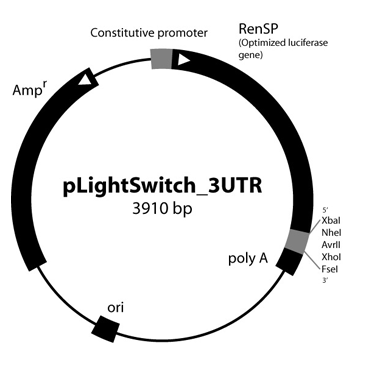


**Fig. S2. The structure of the SOX9 3’UTR encoding reporter plasmid**. The SOX9 3’UTR sequence was cloned into pLightSwitch (pLS) plasmid via NheI/XhoI digestion. The resulting reporter plasmid was then used to verify direct interactions between the miRNAs under investigation and the SOX9 3’UTR via luciferase assay.

**A B**


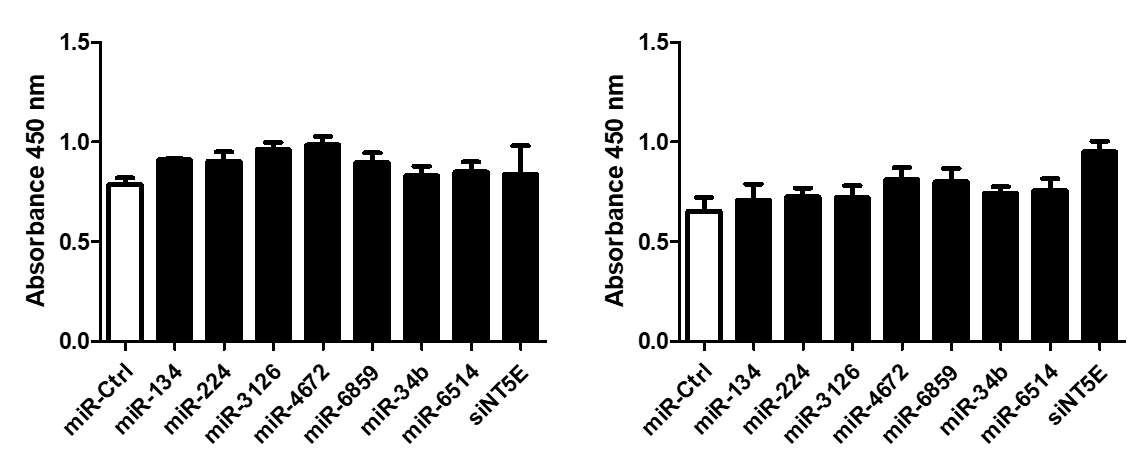


**Fig. S3. XTT assay using MDA-MB-231 (A) and MCF-7 (B) cells**. Cell Viability was monitored by XTT assays using the same conditions as applied during the luciferase reporter assay to exclude potential interference of results caused by miRNA mediated effects on cell viability. Four transfections were performed per condition. Data is shown as Mean ± SD. No significant changes in viability were observed for the condition applied during the reporter assays.

**Fig. S4. Independent repetitions of luciferase SOX9 3’-UTR reporter assay.** MDA-MB-231 or MCF-7 cells were transfected with 50 nM miRNA and luciferase activity was assessed 24 h later by luminescence measurement. Five transfections were performed per condition. Data is shown as Mean ± SD. Significance was assessed by one-way ANOVA using Dunnett’s multiple comparison test. All samples were compared to mimic control-1 samples. *: p < 0.05; **: p < 0.01; ***: p < 0.001; ****: p < 0.0001.

**Fig. S5. miRNA mediated effects on SOX9 expression in MDA-MB-231 cells determined by microarray analysis.** MDA-MB-231 cells were transfected with 50 nM miRNA and 48 h post transfection cells were harvested and used for subsequent gene expression profiling. Three transfections were performed for each condition. Data is shown as Mean ± SD. Significance was assessed by one-way ANOVA using Dunnett’s multiple comparison test. All samples were compared to mimic control-1 samples. *: p < 0.05; **: p < 0.01; ***: p < 0.001; ****: p < 0.0001.


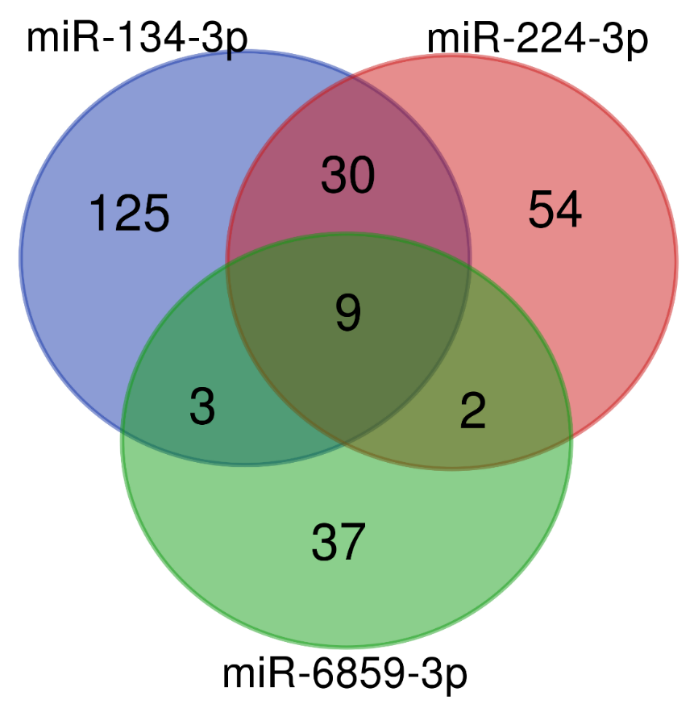


**Fig. S6. Venn diagram of genes showing decreased gene expression upon transfection of miR-134-3p, miR-224-3p and miR-6859-3p.** MDA-MB-231 cells were transfected with 50 nM miRNA and subsequent gene expression profiling was performed. Genes with significantly decreased expression levels compared to mimic control-1 transfections with a fold change < 0.5 were considered.


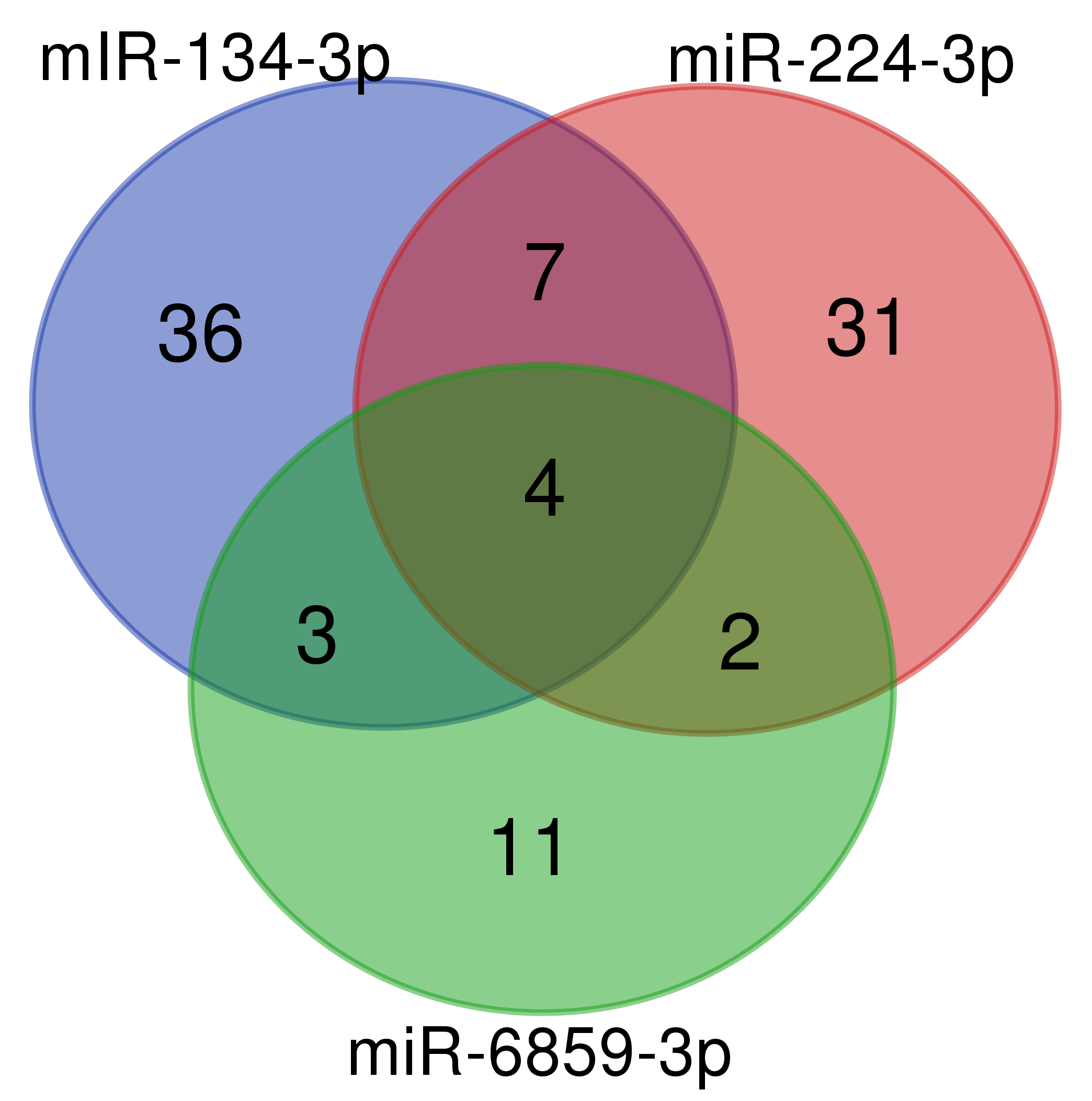


**Fig. S7. Venn diagram of significantly enhanced genes**. MDA-MB-231 cells were transfected with 50 nM miRNA and subsequent gene expression profiling was performed. All genes significantly enhanced compared to mimic control-1 transfections with a fold change > 2 were considered.

**
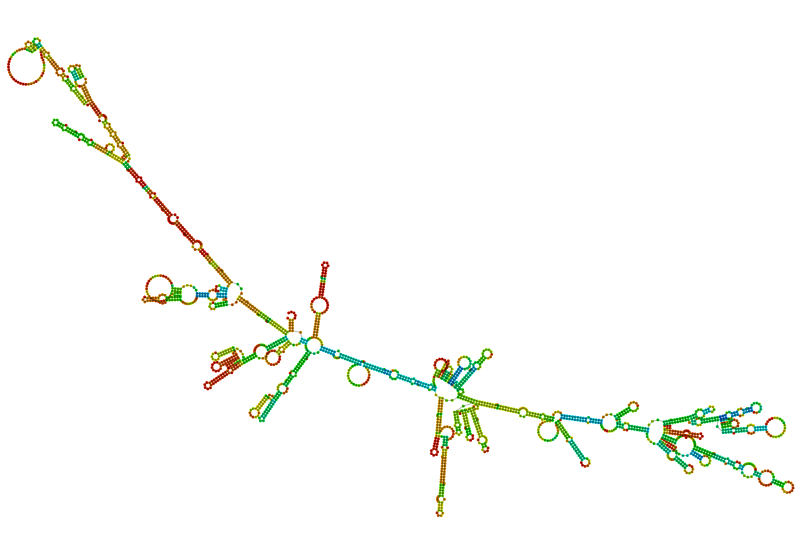
**

**Fig. S8. RNA fold prediction of SOX9 3’-UTR sequence**. The Minimum Free Energy (MFE) structure of SOX9 3’-UTR sequence was retrieved with RNAfold WebServer (<http://rna.tbi.univie.ac.at/cgi-bin/RNAWebSuite/RNAfold.cgi>). As input sequence we used the provided DNA sequence of the SOX9 3’-UTR within the pLS plasmid converted to mRNA sequence by Nucleic Acid Converter tool (<https://skaminsky115.github.io/nac/index.html>).


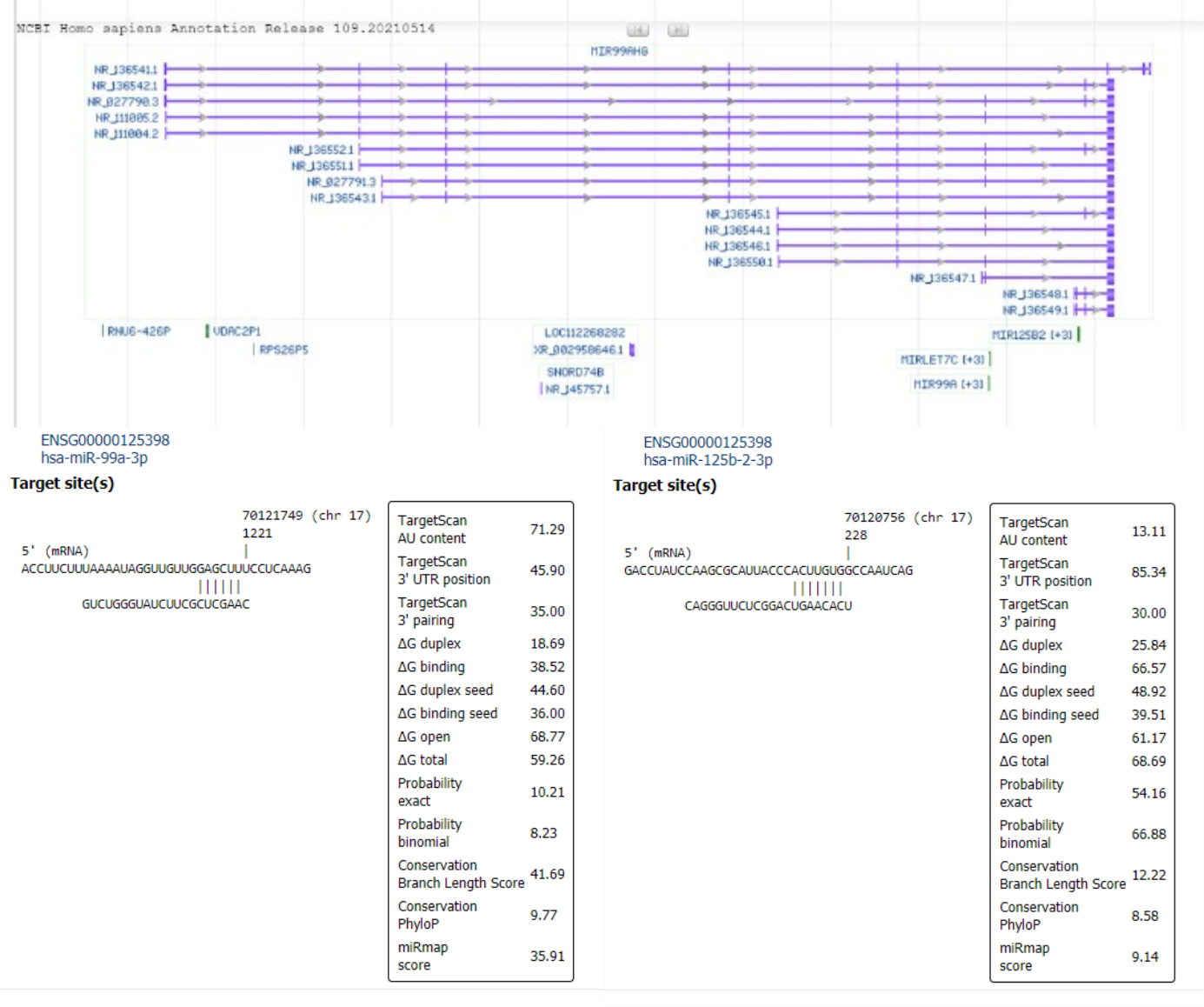


**Fig. S9. MIR99AHG was significantly enhanced upon miR-6859-3p transfection in MDA-MB-231 cells.** MIR99AHG was among the significantly up-regulated genes (FC = 1.61, p = 0.009) based on Microarray gene expression profiling in MDA-MB-231 cells transfected with miR-6859-3p compared to mimic control-1 transfection. This gene encodes miR-let-7c, miR-99a and miR-125b-2 miRNAs. miR-99a-3p and miR-125b-2-3p are both predicted to likely bind to SOX9 3’-UTR. Gene information was retrieved from NCBI (<https://www.ncbi.nlm.nih.gov/gene/388815>) and miRNA binding information was retrieved from miRmap tool (https://mirmap.ezlab.org/app/).

**
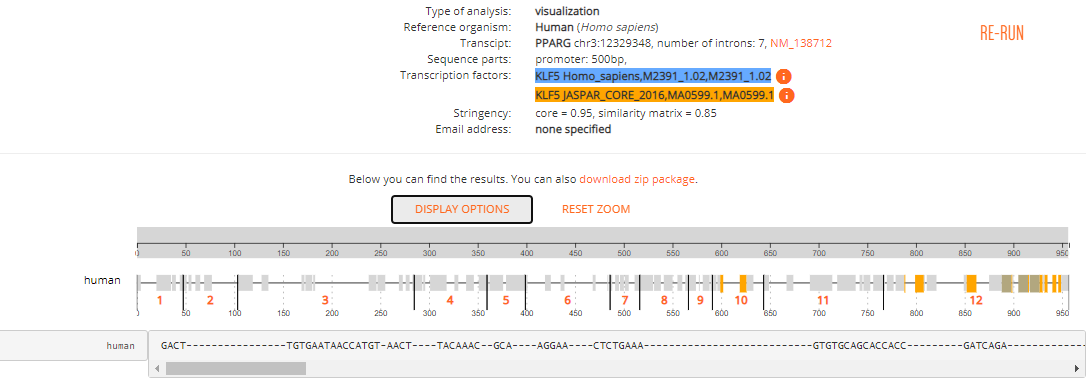
**
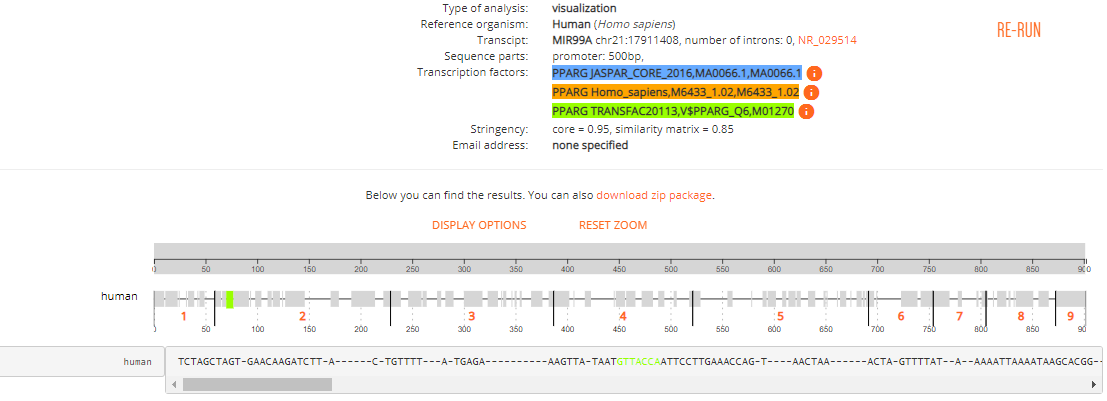
**
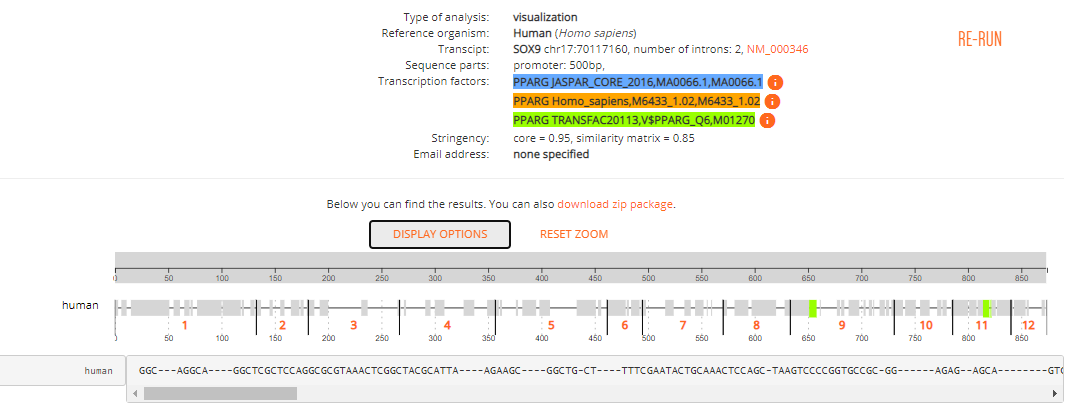
**

**Fig. S10. PPARG is negatively correlated with MIR99AHG mRNA levels but positively correlated with SOX9 mRNA levels**. To find a possible explanation for observed MIR99AHG up-regulation upon miR-6859-3p transfection, we searched for transcription factors that are significantly negatively correlated with MIR99AHG mRNA levels within the NCI-60 data set [1] (PCC < - 0.35) and that were inhibited by miR-6859-3p transfection in our MDA-MB-231 Microarray data (FC < 0.7, p < 0.05). We found PPARG to be negatively correlated with MIR99AHG mRNA levels (PCC = -0.39, p = 0.0022) and interestingly, positively correlated with SOX9 mRNA levels (PCC = 0.40, p = 0.0015). The promoter regions of the MIR99AHG and SOX9 encoding genes exhibit PPARG binding sites. Furthermore, PPARG expression levels were significantly reduced upon miR-6859-3p transfection (FC = 0.70, p = 0.03). miR-6859-3p is no predicted binder of the PPARG 3’-UTR, but carries a potential binding site for the KLF5 3’-UTR. KLF5 is a known transcriptional activator of PPARG and the PPARG promoter contains several PPARG binding sites [2, 3].

**
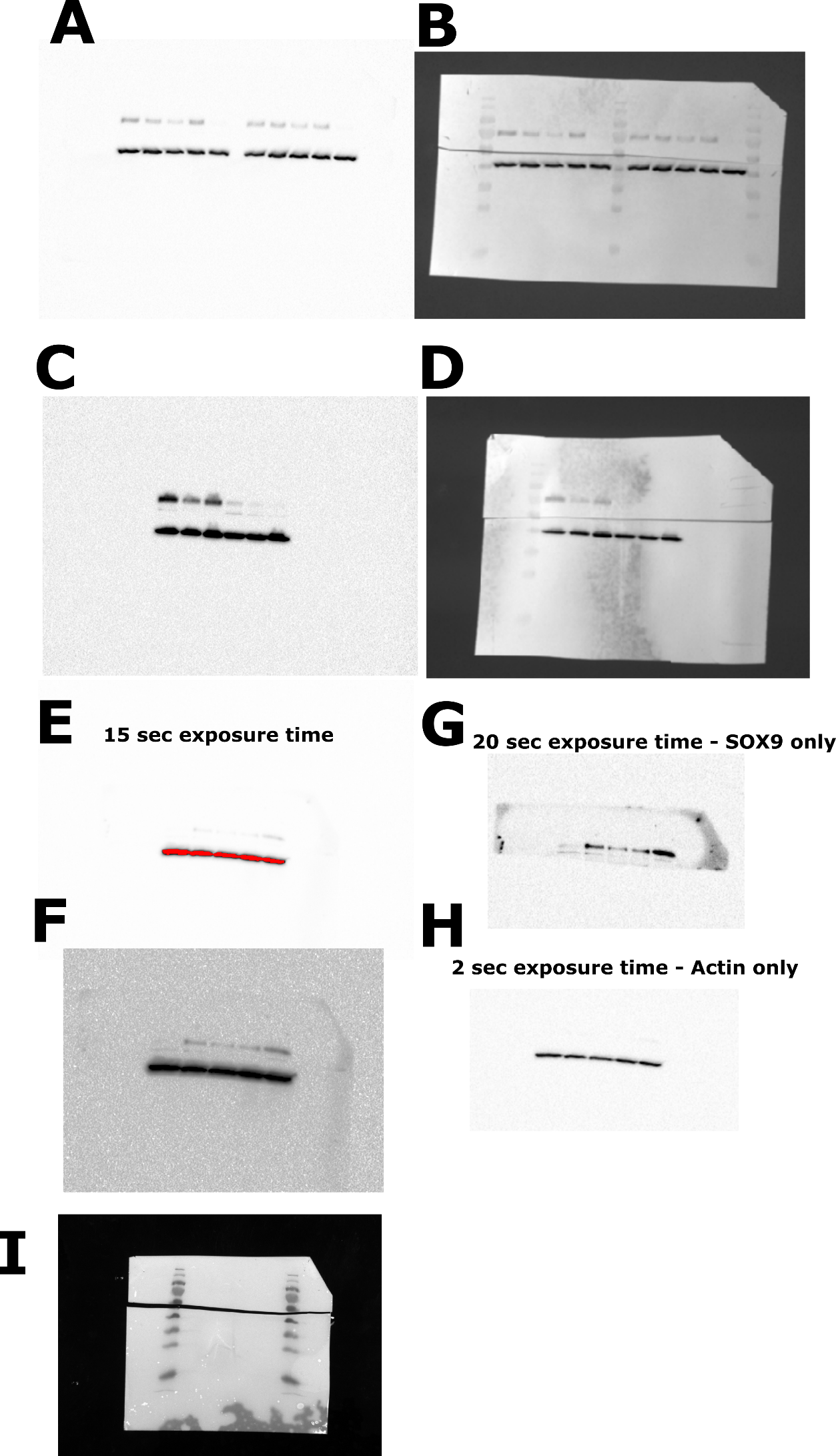
**

**Fig. S11. Full-size images of Western blots used in this study**. Procedure: After transfer of separated proteins, membranes were cut into two pieces enabling independent incubation of the upper and the lower membrane part with SOX9 and β-actin specific antibodies, respectively. Subsequently, both blots were positioned and aligned in the imager a photograph was taken to identify markers, chemiluminescence was measured, and both pictures were overlaid. **(A, B)** Full-size image of the Western blot shown in Figure 1C. Sample order is as indicated in Figure 1C. Chemiluminescence was determined (A) at an exposition time of 0.5 seconds and merged with a photograph of the blot (B). **(C, D)** Full-size image of the Western blot used to quantify changes in SOX9 protein levels after miRNA transfection as depicted in Figure 1B. Chemiluminescence detection (C) was performed with an exposition time of 0.5 seconds followed by overlay with the corresponding photograph (D). Sample order: mimic control-1, miR-134-3p, miR-6514-3p, SOX9 siRNA 12.5 nM, SOX9 siRNA 25 nM and SOX9 siRNA 50 nM. **(E-I)** Full-size images of an independent Western blot replicate used for quantification shown in Figure 1B. Simultaneous detection of SOX9 and β-actin with an exposition time of 15 seconds resulted in oversaturated β-actin signals (E). Membrane contours became visible by variation of the high, low and gamma settings (F). Subsequently, the SOX9-specific signal was measured separately at an exposition time of 20 seconds (G) or of 2 seconds in case of β-actin (H). Sample order: SOX9 siRNA, miR-6859-3p, miR-224-3p, miR-134-3p and mimic control-1. As the camera for the colorimetric photograph was malfunctioning at the time of the experiment, a later picture was taken from the excised plots (I), which could not be used for an overlay.

**
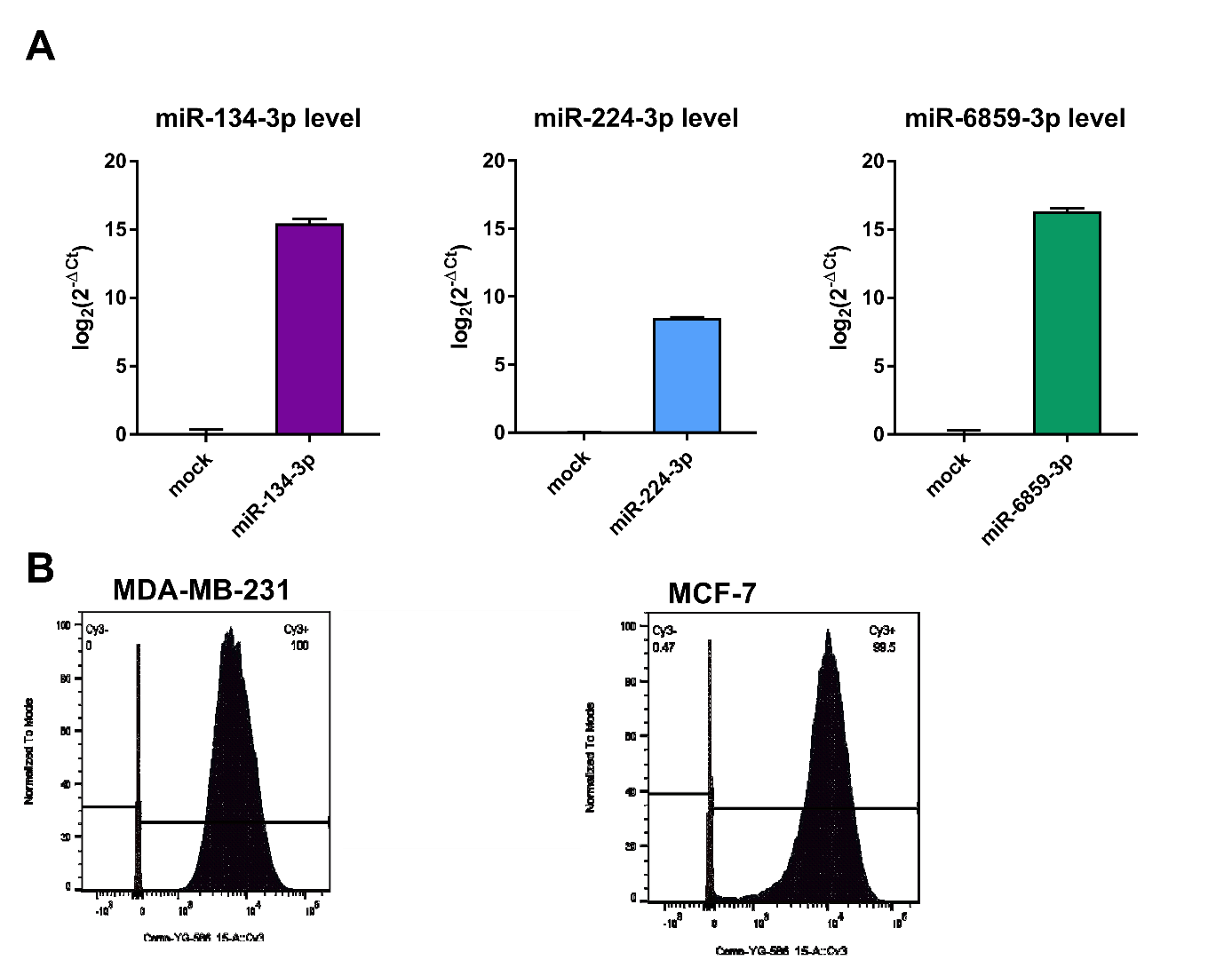
**

**Fig. S12. Efficacy of miRNA mimic transfection**. MDA-MB-231 cells (A+B) or MCF-7 cells (B) were transfected with 50 nM miR-134-3p, miR-224-3p, miR-6859-3p or miR-Cy3. 48 h post transfection cells were harvested for **(A)** RNA isolation followed by qPCR to assess cell intracellular miRNA levels using specific taqman probes or **(B)** for flow cytometry analysis of percentage of Cy3+ cells.

**Fig S13. Dose Standardization**. MDA-MB-231 cells were transfected with 12.5, 25 50 or 100 nM miR-134-3p, miR-224-3p, miR-6859-3p or SOX9 siRNA pool. 48 h post transfection, cells were harvested for RNA isolation followed by qPCR to determine SOX9 mRNA levels. RPL19 was used as house keeping gene. Samples were normalized to mock transfected cells.

**References**

1. Reinhold WC, Sunshine M, Liu H, Varma S, Kohn KW, Morris J, Doroshow J, Pommier Y: **CellMiner: a web-based suite of genomic and pharmacologic tools to explore transcript and drug patterns in the NCI-60 cell line set**. *Cancer Res* 2012, **72**(14):3499-3511.

2. Lee JE, Ge K: **Transcriptional and epigenetic regulation of PPARgamma expression during adipogenesis**. *Cell Biosci* 2014, **4**:29.

3. Oishi Y, Manabe I, Tobe K, Tsushima K, Shindo T, Fujiu K, Nishimura G, Maemura K, Yamauchi T, Kubota N *et al*: **Kruppel-like transcription factor KLF5 is a key regulator of adipocyte differentiation**. *Cell Metab* 2005, **1**(1):27-39.
